# Three-Dimensional Printable Enzymatically Active Plastics

**DOI:** 10.1021/acsapm.1c00845

**Published:** 2021-11-15

**Authors:** William H. Zhang, Graham J. Day, Ioannis Zampetakis, Michele Carrabba, Zhongyang Zhang, Ben M. Carter, Norman Govan, Colin Jackson, Menglin Chen, Adam W. Perriman

**Affiliations:** † School of Cellular and Molecular Medicine, University of Bristol, Bristol BS8 1TD, United Kingdom; ‡ Bristol Composites Institute (ACCIS), University of Bristol, Bristol BS8 1TR, United Kingdom; § Bristol Medical School, Translational Health Sciences, University of Bristol, Bristol BS2 8DZ, United Kingdom; ∥ Interdisciplinary Nanoscience Center (iNANO), Aarhus University, Aarhus DK-8000, Denmark; ⊥ Defence Science and Technology Laboratory, Porton Down, Salisbury SP4 0JQ, United Kingdom; # Australian National University, Research School of Chemistry, Canberra ACT 2601, Australia; ∇ Australian Research Council Centre of Excellence for Innovations in Peptide and Protein Science, Research School of Chemistry, Australian National University, Canberra, ACT 2601, Australia; ○ Australian Research Council Centre of Excellence in Synthetic Biology, Research School of Chemistry, Australian National University, Canberra, ACT 2601, Australia

**Keywords:** nanocomposite, nanomorphology, functional bionanomaterials, enzyme, nanoconjugate, 3D printing, melt electrowriting

## Abstract

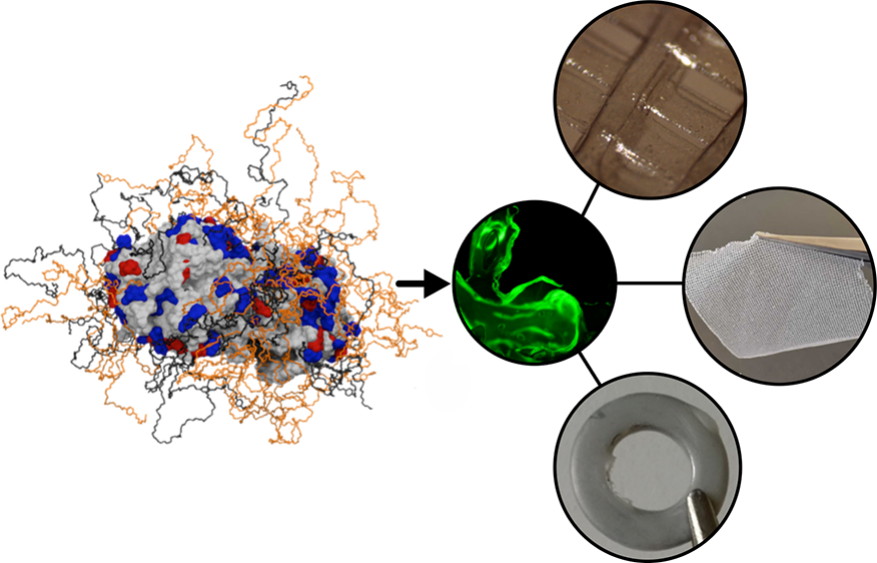

Here, we describe a facile route to the synthesis of enzymatically active highly fabricable plastics, where the enzyme is an intrinsic component of the material. This is facilitated by the formation of an electrostatically stabilized enzyme–polymer surfactant nanoconstruct, which, after lyophilization and melting, affords stable macromolecular dispersions in a wide range of organic solvents. A selection of plastics can then be co-dissolved in the dispersions, which provides a route to bespoke 3D enzyme plastic nanocomposite structures using a wide range of fabrication techniques, including melt electrowriting, casting, and piston-driven 3D printing. The resulting constructs comprising active phosphotriesterase (arPTE) readily detoxify organophosphates with persistent activity over repeated cycles and for long time periods. Moreover, we show that the protein guest molecules, such as arPTE or sfGFP, increase the compressive Young’s modulus of the plastics and that the identity of the biomolecule influences the nanomorphology and mechanical properties of the resulting materials. Overall, we demonstrate that these biologically active nanocomposite plastics are compatible with state-of-the-art 3D fabrication techniques and that the methodology could be readily applied to produce robust and on-demand smart nanomaterial structures.

## Introduction

Functional bionanomaterials comprising enzymes and synthetic polymers provide an attractive opportunity to increase the diversity of chemical milieu encountered by protein-based components. This is because the chemical construction of a surface-bound polymer surfactant corona enables biomolecules to be utilized in a range of dielectric media and, potentially, in both the solid and liquid phases. Here, the polymer surfactant corona serves two primary purposes. It provides an interface with a polarity that is compatible with the desired dispersion media, and it supports correct folding of the protein. Moreover, a well-designed corona has the potential to even improve the performance of enzymes by increasing the rate of substrate and product transport by varying the dielectric constant in the vicinity of the active site.^1^

Our previous work has shown that discrete hybrid macromolecules comprising electrostatically stabilized protein–polymer surfactant nanoconjugates with well-defined stoichiometries can be used to generate a raft of biologically active materials with emergent properties. For example, the bioconjugates can be dehydrated to produce solvent-free liquid proteins with oxygen-binding properties,^2^ hierarchically self-assembled to produce porous membranes with recyclable catalytic activity,^3−5^ or partitioned into hydrophobic cell membrane domains to yield artificial membrane-binding proteins.^6−8^ Significantly, the proteins in these hybrid materials are folded, biologically active, hyper-thermostable (*T_m_* = 155 °C),^9^ and have protein dynamics that closely resembled those of fully hydrated proteins.^10−12^ Moreover, using this approach, we developed a new self-contained enzymatic biofluid, where lipases were re-engineered to produce room temperature liquids that required no dispersion medium, could solubilize substrates, and catalyze the hydrolysis of fatty acid esters up to temperatures of 150 °C.^13^

Rationally modifying enzymes such that they could be utilized in heterogeneous catalysis is an attractive prospect. Accordingly, there has been a significant research effort focused on the incorporation of enzymes into functional solid materials through surface immobilization^14−16^ or *via* the development of biomimetic hydrogels.^17^ Surface immobilization has allowed enzymes to be incorporated as persistent components of flow reactors, resulting in increased product yields,^18^ and these immobilization methods can also be combined with quantum-dot technologies to generate enzymatically active quantum dot fibres.^19^ Biomimetic hydrogels show immense promise in biomedical applications, facilitating the slow release of bioactive enzymes such as RNases, globulins,^20^ or antibodies,^21^ and can provide a bioresorbable 3D scaffold that promotes wound healing.^22,23^ However, despite the advances in solid-state enzymolysis, there are still key limitations that reduce their widespread industrial utility. For example, surface-immobilized enzymes may be denatured, depleted, or fouled and thus require a tailored environment to preserve surface activity.^14^ Moreover, enzymes immobilized in hydrogel matrices typically have limited mechanical and environmental stability and can require specific conditions to retain their gel phase,^24^ meaning that their application is generally restricted to controlled biomedical environments.^25−27^ A significant improvement would therefore be to successfully integrate enzymes into more ubiquitous structural materials that have widespread utility.

An attractive alternative to gel integration or surface immobilization is to re-engineer the surface of an enzyme such that it is readily dispersible in a hydrophobic medium, which would provide a route to integration with a suitable material feedstock for the fabrication of “smart” solid-state structures; materials able to autonomously perform specific functions in response to environmental stimuli or substrates. In this scenario, the enzyme exists as an integral component within the material, which could even present new active surfaces through tunable material degradation profiles. There are a number of reported examples of enzymes that retain their native structure and catalytic activity in organic solvents and ionic liquids when enclosed in a sheath of polymers,^28−32^ which opens up exciting new possibilities in the field of smart material fabrication. Until recently, there have only been very few examples of enzymes being utilized as integrated components in plastics, with the closest examples being the fabrication of enzyme-rich cross-linked membranes,^4^ enzyme–silk composites,^33^ or enzymes adsorbed onto plastics through electrospinning.^34^ However, there have been great strides in regards to the integration of enzymes, with significant focus on the incorporation of lipases within plastics to generate impressive self-biodegrading materials.^35−38^ Collectively, these studies have exploited the promiscuous hydrolytic capacity of lipases toward polymer chains, where in the presence of a suitable catalytic environment (usually buffered water), the catalysis of the plastic polymers occurs rapidly within days. Excitingly, the scope of possible functionalities has expanded further, where exogenous substrate catalysis has also shown to be possible, where recently Xu and colleagues used random heteropolymers (RHP) to encapsulate the enzyme organophosphorus hydrolase (OPH) and provide stability in toluene for polycaprolactone (PCL) film and fiber formation. Here, the authors demonstrated that the reusable and robust OPH-loaded fiber mats were capable of degrading organophosphates over a period of 3 months with residual activities of at least 40%. Such materials have immense promise as a means of bioremediation, as pollutants such as organophosphates persist for long periods of time in the environment and cause long term adverse health effects and deaths.^39^ Such compounds have lipophilic properties and are known to sequester into similarly composed materials, such as polymer paint coatings or tar roads,^40^ and as such, imbuing these types of materials with self-decontaminating properties would counteract such sequestration.

Within the rapidly emerging field of smart nanostructures and devices, demonstrating the compatibility of these smart hybrid materials with modern high-resolution fabrication techniques is key for their adoption as a viable and practical material. Accordingly, we show that enzymatically active plastics can be readily fabricated into high-resolution 3D structures on demand using piston-driven 3D (PD3D) printing and thermal extrusion methods, including melt electrowriting (MEW). These enzyme plastics retain function, with catalytic activity persisting across multiple assays and throughout prolonged exposure to an aqueous environment. As we have previously shown that this protein surface reengineering methodology is widely applicable across different structural and evolutionary families of enzymes,^1^ the approach could be readily adopted using a wide range of fabrication techniques to produce bespoke solid structures with complex chemistries.

## Results and Discussion

**Figure 1:**
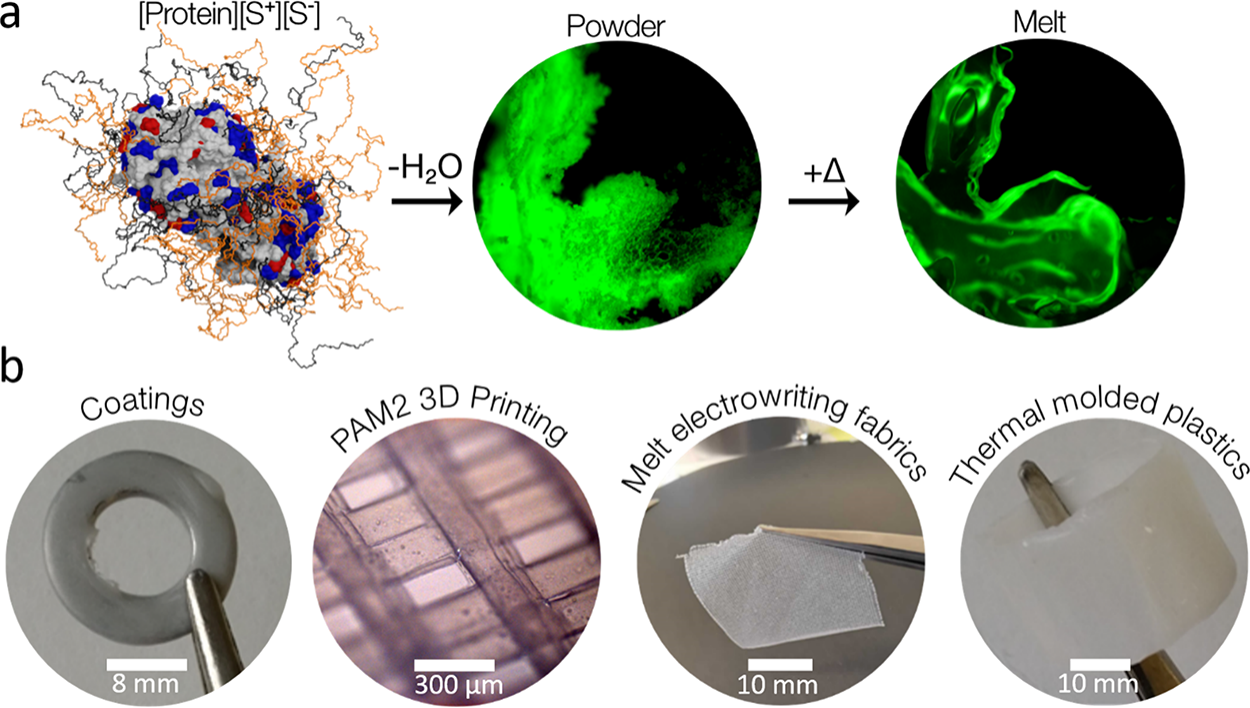
(a) Schematic demonstrating the formation of the solvent-free protein melt. The surface of the enzyme (arPTE) or protein (sfGFP) is modified *via* the sequential addition of cationic and anionic polymer surfactants and is then lyophilized to create a dry powder. Upon heating, the powder melts form a solvent-free liquid. Crucially, this does not inhibit the biological function, as shown by the retention of sfGFP fluorescence. (b) The resulting protein melts can be co-solubilized with plastic precursors in organic solvents to provide access to a range of enzymatically active plastics, including active coatings, piston-driven 3D (PD3D) printed structures, membrane fabrics from melt electrowriting (MEW), and large monoliths from thermal molding.

Using our recently reported sequential electrostatic addition strategy, which provides a route to functional bioconjugates without the need for covalent protein modifications or mutagenesis (Figure 1a),^1^ we produced polymer surfactant nanoconjugates of phosphotriesterase from *Agrobacterium radiobacter* (arPTE),^41^ as well as superfolder green fluorescent protein (sfGFP).^42^ The resulting aqueous protein–polymer surfactant nanoconstructs were then lyophilized and thermally annealed to form solvent-free protein “melts” (Figure 1a), termed [arPTE][S^+^][S^–^] and [sfGFP][S^+^][S^–^], respectively. Differential scanning calorimetry (DSC) performed on the anhydrous liquids showed an endothermic melting transition at 33.9 ± 0.1 °C for [arPTE][S^+^][S^–^] and 33.0 ± 0.04 °C for [sfGFP][S^+^][S^–^] (Figure S1). Temperature-dependent synchrotron radiation wide-angle X-ray scattering (WAXS) experiments performed on the [arPTE][S^+^][S^–^] showed that the endothermic transition observed in the DSC was commensurate with a loss of the crystalline features at *q*-values of 1.34 and 1.63 Å and a midpoint melting transition temperature of approximately 33.6 °C (Figure S2). These features correspond to the PEG–PEG chain and nonylphenyl–nonylphenyl tail interaction distances^43^ and support the transition from a semi-crystalline solid to an amorphous fluid. Temperature-dependent synchrotron radiation–circular dichroism spectroscopy showed a significant increase in the thermal stability of the [arPTE][S^+^][S^–^] melt (*T_m_* = 102.4 ± 2.0 °C), when compared with the native arPTE enzyme (*T_m_* = 71.2 ± 0.6 °C; Figure S3).

**Figure 2:**
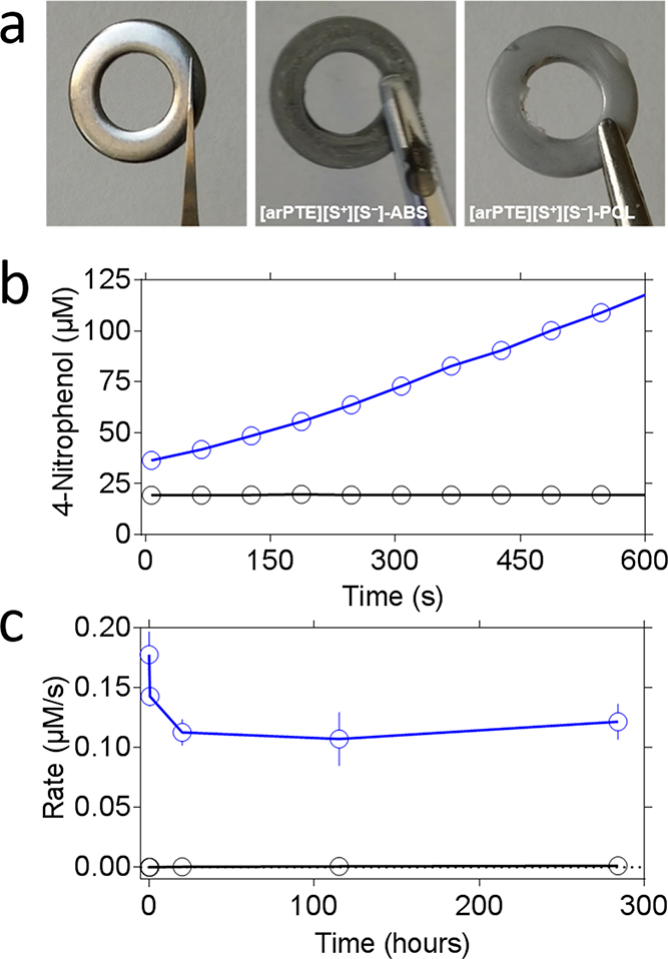
(a) A 20 mm-diameter stainless steel metal washer before (left) and after evaporative coating from acetone containing 1% w/v [arPTE][S^+^][S^–^] and 10% w/v acrylonitrile butadiene styrene (ABS) (middle), and chloroform containing 1% w/v [arPTE][S^+^][S^–^] and 10% w/v polycaprolactone (PCL) (right). (b) Paraoxon hydrolysis activity of [arPTE][S^+^][S^–^]–ABS-coated rings (blue) compared to the activity of enzyme-free control rings (black). (c) Activity of the [arPTE][S^+^][S^–^]–ABS coated rings over an extended time period across multiple assays to assess lifetime and reusability. The exact same ring was used for the series of assays with persistent exposure to an aqueous environment between assays.

The viscous anhydrous protein melts could be readily dispersed in a range of organic solvents, including acetone, tetrahydrofuran, acetonitrile, and chloroform (Figure S4). Acetone was used to produce co-dispersions of [arPTE][S^+^][S^–^] and acrylonitrile butadiene styrene (ABS) and chloroform for analogous formulations in PCL. PCL was of special interest as it is biocompatible, biodegradable, and can be thermally extruded or molded at lower temperatures (*ca.* 60 °C) than many other plastics.^44,45^ Removal of the solvent from these co-dispersions gave solid enzymatically active plastic materials. To demonstrate the utility of the approach, the enzyme plastics were applied as solid film coatings to metals, 3D printed using piston-based extrusion, or directly fabricated from the bulk material using thermal extrusion or molding (Figure 1b). Evaporative coating was the simplest method of preparation, where a solid template could be dip-coated or painted with the enzyme–plastic dispersion (Figure 2a), and multiple applications could be applied to increase the layer thickness of the enzymatically active coatings. [arPTE][S^+^][S^–^]–ABS-coated rings also exhibited organophosphate hydrolase activity (Figure 2b), and the material showed an asymptotic decline in activity (Figure 2c) over 300 h that plateaued after 24–48 h.

**Figure 3:**
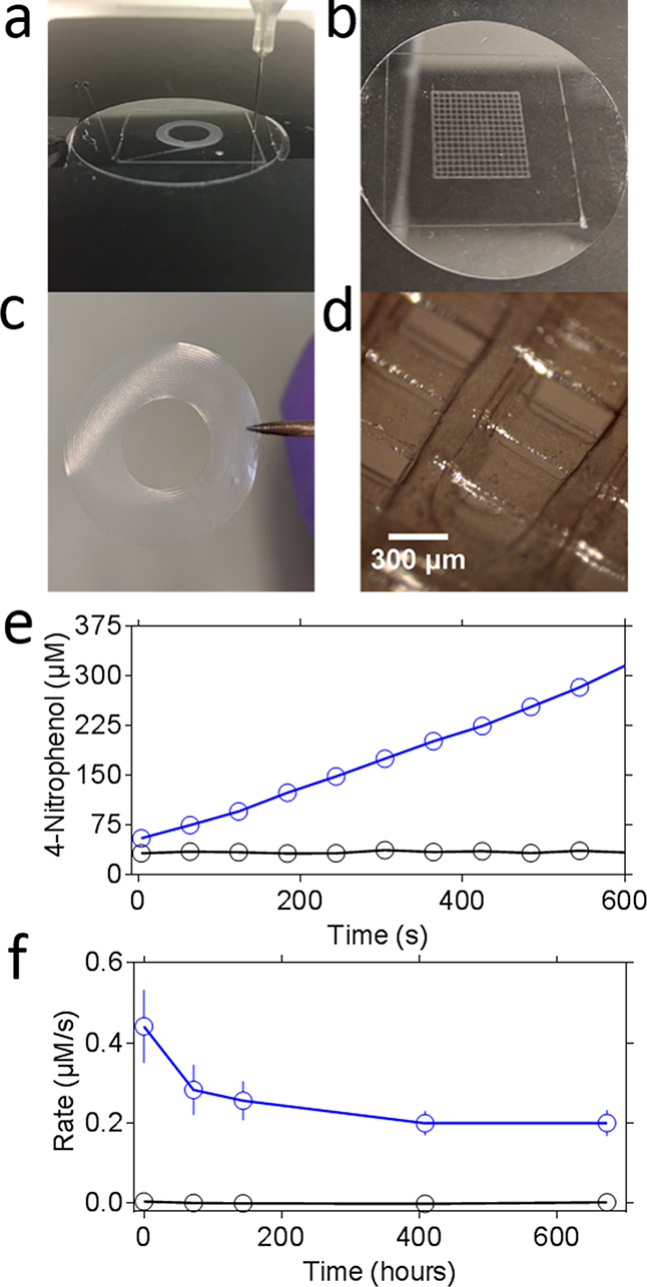
(a) Example of piston-driven 3D (PD3D) printing, where a mixture of PCL, enzyme, and solvent ([arPTE][S^+^][S^–^]–PCL in chloroform 10–15% w/v) can be extruded through a piston-driven printing system to create structures such as multilayered rings. As with the coatings, enzyme loading was at 1% w/w enzyme/plastic. (b) An example of a multilayer 3D woodpile structure that can be created using this fabrication method, 1.5 cm^2^ in size. (c) An example of a multilayer ring also created with the PD3D method, 2 cm in diameter. (d) A close-up view of the woodpile structure printed with PD3D printing, showing 3D layering. (e) Paraoxon hydrolysis activity of [arPTE][S^+^][S^–^]–PCL rings (blue) compared to the activity of enzyme-free control rings (black). (f) The activity of the [arPTE][S^+^][S^–^]–PCL solvent-printed rings over an extended time period across multiple assays to assess lifetime and re-usability. As with the coated rings, the exact same ring was used for the series of assays with persistent exposure to an aqueous environment between assays.

This activity profile was mirrored in plastic structures fabricated using PD3D printing of the [arPTE][S^+^][S^–^]–PCL chloroform dispersions.^46−48^ This method allows the controlled deposition of the low-viscosity solution, enabling structures such as discs and woodpiles to be reliably printed (Figure 3a–d). We focused on the use of PCL with these more advanced fabrication techniques, as ABS lacks the biodegradable and biocompatible properties of PCL, and otherwise requires much higher temperatures for thermal printing. As with the coated rings, 3D-printed [arPTE][S^+^][S^–^]–PCL rings (1% enzyme w/w) both retained significant activity and exhibited an asymptotic decline and plateau of enzymatic activity (Figure 3e,f), with this plateau of activity persisting beyond 650 h. PD3D printing was also used to produce 3D structures with the same volumes but increased surface areas. In practice, this was performed by printing ring structures with varying surface areas and layer numbers (Figure S5). Interestingly, the activity did not correlate with the external surface area but rather with the number of printed layers (Pearson’s *r* = 0.99; Figure S6). Scanning electron microscopy (SEM) images from delaminated multilayer structures revealed high levels of interlayer roughness (Figure S7). Accordingly, it is likely that the correlation between the activity and layer number resulted from the increase in the reaction surface area from these high surface area interlayer structures.

**Figure 4:**
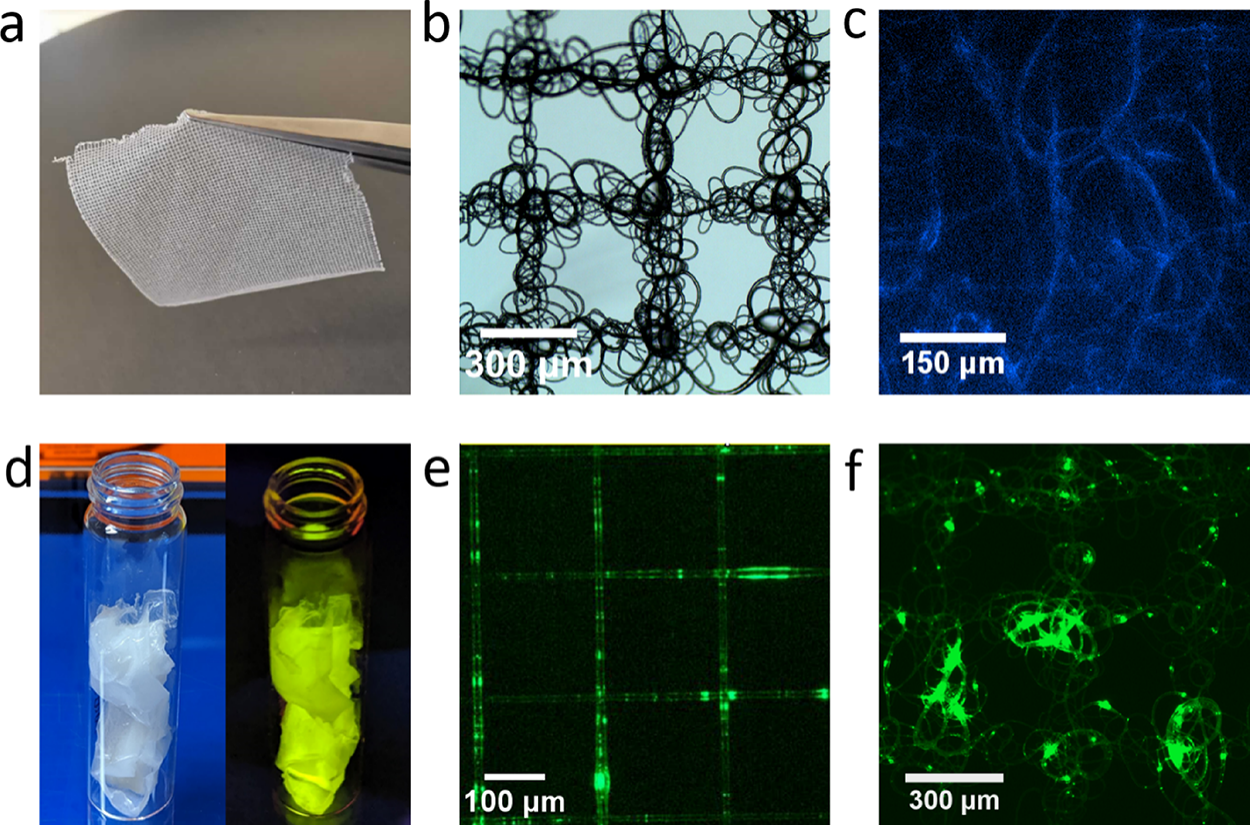
(a) Melt electrowriting allows for the PCL to be printed as fine threads, which can be used to create active enzyme–PCL fabrics ([arPTE][S^+^][S^–^]–PCL). The fabric shown is 3 cm^2^ in size. (b) Close examination of the [arPTE][S^+^][S^–^]–PCL fabric with widefield microscopy show that it is composed of tight tangles of enzyme–PCL threads arranged in a discrete repetitive pattern (fibers, <5 μm in thickness). (c) Enzymatic activity of the material is retained after the melt electrowriting process, as shown through widefield fluorescence microscopy of the fabric in the presence of Coumaphos, a phosphothioate that is hydrolyzed by arPTE into the fluorescent product chlorferon (excitation wavelength 355 nm, emission peak 460 nm). (d) sfGFP can similarly be infused into PCL in the same manner as arPTE, to create fluorescent plastics ([sfGFP][S^+^][S^–^]–PCL). Shown here is the resulting solid bulk material under normal lighting (left) and when illuminated with blue light (right). (e) The [sfGFP][S^+^][S^–^]–PCL material can be printed to create fine fluorescent structures such as the ordered grid shown. (f) The [sfGFP][S^+^][S^–^]–PCL material can similarly be used to create fabrics, with the same ordered repetitive tangles as previously shown with [arPTE][S^+^][S^–^]–PCL.

The [arPTE][S^+^][S^–^]–PCL enzyme plastics could also be directly fabricated using thermal extrusion or molding of the solid matrix after removal of the solvent. This provided access to 3D printer filaments and thermally molded monoliths, allowing for the fabrication of larger structures (Figure S8). Moreover, the high-resolution 3D fabrication technique MEW could be used to produce precise micrometer resolution structures from [arPTE][S^+^][S^–^]–PCL and [sfGFP][S^+^][S^–^]–PCL, such as fabric meshes with ultrafine threads (<5 μm, 0.1% enzyme w/w; Figure 4).

**Figure 5:**
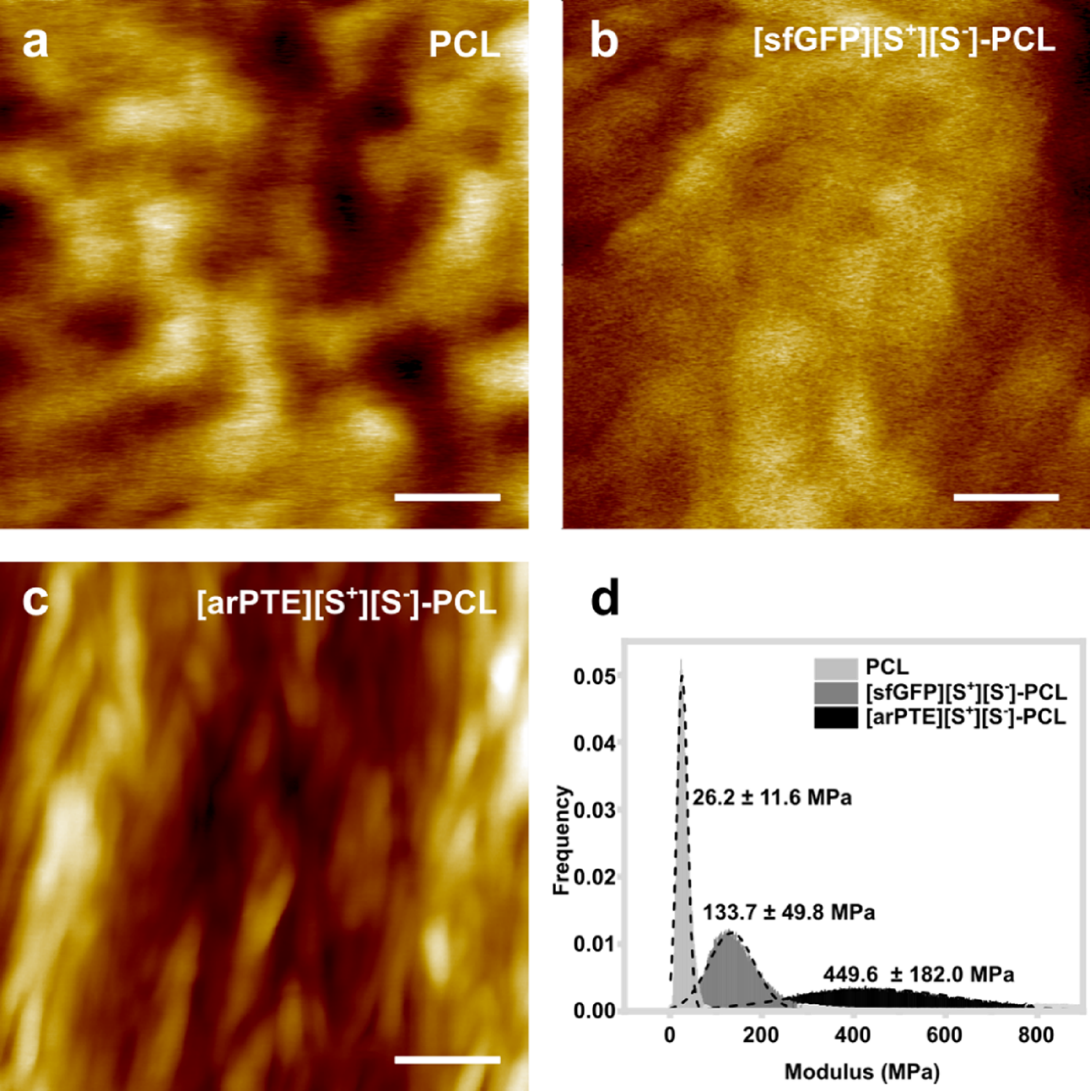
Topographical morphology of melt electrowritten fibers from AFM analysis of (a) PCL, (b) [sfGFP][S^+^][S^–^]–PCL, and (c) [arPTE][S^+^][S^–^]–PCL, where [arPTE][S^+^][S^–^]–PCL shows fiber alignment that is not present in the PCL topography. Scale bar, 200 nm. (d) Histograms of Young’s modulus with Gaussian fittings obtained from PCL, [sfGFP][S^+^][S^–^]–PCL, and [arPTE][S^+^][S^–^]–PCL showing a progressive increase in stiffness.

Quantitative nanomechanics (QNM) mapping of the MEW plastic fibers was used to investigate the impact of the protein guest identity (arPTE or sfGFP) on the morphology and mechanical properties of the [arPTE][S^+^][S^–^]–PCL and [sfGFP][S^+^][S^–^]–PCL. Here, nanoscale topographical morphology mapping and nanoindentation experiments were performed and the Young’s moduli were evaluated for three different materials (PCL, [arPTE][S^+^][S^–^]–PCL, and [sfGFP][S^+^][S^–^]–PCL; Figure 5). When compared with the neat PCL (Figure 5a), both [sfGFP][S^+^][S^–^]–PCL (Figure 5b) and [arPTE][S^+^][S^–^]–PCL (Figure 5c) appeared to exhibit morphologies with increased levels of ordered structure. This was particularly apparent in the [arPTE][S^+^][S^–^]–PCL plastic, with highly aligned fibrillar structures perpendicular to the extrusion axis, which is indicative of high-order molecular orientation and crystallinity. The differences in the nanoscale morphology also impacted on the nanomechanical properties of the materials. Here, the QNM maps (Figure S9) were used to determine the Young’s modulus through DMT model fitting, and the overall statistical average modulus of these materials were found to be 26.2 ± 11.6, 133.7 ± 49.8, and 499.6 ± 182.0 MPa for PCL, [sfGFP][S^+^][S^–^]–PCL, and [arPTE][S^+^][S^–^]–PCL, respectively (Figure 5d).

To investigate whether the variation in the Young’s moduli could be reconciled with either a change in the degree of crystallinity or lattice arrangement in the two different protein-based plastics, differential scanning calorimetry (DSC) was used to map the crystallization transition temperature (*T_C_*) and melting temperature (*T_m_*) of the enzyme plastics (Figure S10). Significantly, the increase in the Young’s modulus of the samples corresponded to an increase in *T_C_* (30.2 ± 0.1 °C for PCL, 30.8 ± 0.2 °C for [sfGFP][S^+^][S^–^]–PCL, and 32.7 ± 0.1 °C for [arPTE][S^+^][S^–^]–PCL at 0.1% enzyme w/w), and this observation is consistent with a change in the crystal lattice arrangement of the plastic.^49^ For *T_m_*, the small increase in the total enthalpy of melting (Δ*H_m_*) of the enzyme plastic also reflects the small increase in the degree of crystallinity. Although modest changes to the degree of crystallinity can result in large changes in the Young’s modulus,^50^ the large magnitude of increase for the Young’s modulus relative to the small change in the degree of crystallinity indicates that the stiffening of the material, in this instance, is unlikely to be a result of a change in the degree of crystallinity. Furthermore, incorporation of the neat surfactants into the PCL plastic ([S^+^][S^–^]–PCL) reduced the *T_C_* but had a comparatively larger effect on total Δ*H_m_*, indicating that the presence of surfactant alone can account for an increase in the degree of crystallinity but did not wholly account for the difference in crystal packing and thus necessitates that the presence of the enzyme has a distinct influence on the observed changes to the physical properties of the material. The increase in the Young’s modulus is thus not adequately accounted for by a change in the degree of crystallinity, contrary to our initial assumptions.

It is therefore likely that the effect is due to a change in the lattice structure or material arrangement adopted by the hybrid material upon cooling, and this is further supported by the presence of nanofibers in the material (Figure S9), which is indicative of higher-order assemblies within the material. Conceptually, it is possible that the enzyme is ionically anchored to multiple surfactant chains that are unidirectionally entangled with the surrounding PCL chains, and this may have a pseudo-cross-linking effect. This would not necessarily increase or decrease the degree of crystallization but would require the packing of the PCL polymers to change. The permanent (plastic/inelastic) displacement of the enzyme nanoconjugate in the “cross-linked” composite material would thus require a greater degree of physical stress compared to the pure material. We stress the speculative nature of this model and recognize that the mechanistic process between the protein structures influencing the crystallization of the overall material is still not known. This observation does, however, open interesting avenues of material modification if alteration of material crystallinity, and therefore stiffness, is of interest for future study.

For the enzyme plastics, the decrease in the enzymatic rate over time likely arises from the reduced substrate or product diffusion from enzymatic active sites at the solution–surface interface of the material. This phenomenon has also been recognized by other surface area-dependent technologies, such as surface or quantum-dot immobilization,^19^ where the effective catalytic rate of the material was lower due to diffusion limitations that are not accounted for in the classical Michaelis–Menten model of enzyme catalysis. For our material, this effect was observed directly using time-lapse widefield fluorescence microscopy of the material with the fluorescence-yielding organothiophosphate substrate coumaphos.^51^ Here, the surface of the fibrous [arPTE][S^+^][S^–^]–PCL material becomes fluorescent, and only after an extended period does the fluorescent product diffuse into the bulk solution (Figure S11), confirming that microenvironmental effects, such as the presence of diffusion layers, influence the effective activity of the material. Moreover, the observed decrease then plateau in activity is unlikely to be due to enzyme liberation through washing, as multiple assays within the same day with rinsing did not show a decrease in activity (Figure S12a). Furthermore, as with the materials developed by DelRe *et al*.,^52^ this decrease is time-dependent, with a dry and unused stored sample showing the same decrease in activity when compared to a sample stored in solution (Figure S12b). This residual activity appears to persist for at least 3 months when stored in solution (Figure S12c), which demonstrates that the material is remarkably robust. It is still not clear what causes this loss of activity with phosphotriesterase; however, it is possible that the enzyme may end up locked into a thermodynamically favorable, but inactive, conformation.^53,54^ As the material is not stored under completely anhydrous conditions, it is possible that residual ambient water is sufficient enough co-crystallize with the material, thereby facilitating the slow transition of the enzyme into the inactive state.^55^ Investigation into this mechanism would require dedicated computational approaches, which is currently beyond the scope of this work.

Additional characterization of the material with SEM was used to assess the degradation of the material. Storage of the enzyme–PCL material for up to 4 weeks in an aqueous environment showed that PCL with 1% w/w enzyme degrades at an accelerated rate compared to unmodified PCL (Figure S13). The observed cavity formation is similar to what has been previously reported for PCL degradation.^56^ This is most likely occurring at sites of high local concentrations of enzyme, which when dissolved or liberated back into aqueous solution, creates a site of structural weakness that will promote erosion at the defect. However, as the observable enzymatic activity remains at a consistent plateau despite the increase in physical cavitation, this would necessitate that the exposure of new surfaces of active enzyme, confirming that the enzyme is distributed throughout the material. PCL with a lower enzyme composition (0.1%) did not exhibit the same rapid decay, confirming that the defect formation is dependent on the enzyme complex loading, and pH measurements of 0.1% [sfGFP][S^+^][S^–^]–PCL and [arPTE][S^+^][S^–^]–PCL stored in pure water did not significantly acidify, indicating that the PCL was not degraded (Figure S14). This maintenance of activity is a distinct advantage, as this increases the functional lifetime of the material in comparison to materials that only have a monolayer of an active component. This particularly promising for the fabrication of medically relevant bioactive implants and tools, where surface-functionalized materials have shown immense success for tissue culture and growth promotion,^57−59^ and the preservation of activity would greatly increase the viable lifetime of implants and implements.

## Conclusions

We have demonstrated that these composite enzyme plastics have high levels of temporal activity retention and can be readily fabricated using 3D printing technologies to create micro- and macroscale structures, both with and without thermal extrusion. These materials also have the capacity for high enzyme loadings to increase total activity. The arPTE-infused plastics can in principle be utilized to develop self-decontaminating surfaces, while the sfGFP-infused plastics may serve as an avenue to develop a fully biodegradable fluorescent plastic, without the need for synthetic dyes. We show that these materials can be exploited through thermally driven 3D-printing methods, allowing for the fabrication of detailed and complex creations such as fabrics. In cases where the thermostability of the enzyme is of concern or the plastic matrix has a high melting temperature, evaporative printing provides an alternative to thermal extrusion to also create complex 3D structures without thermally compromising activity. In principle, this technology is compatible with a wide array of polymeric materials, provided that an appropriate solvent can be used as a medium to generate the enzyme plastic composite. Furthermore, as these materials are surface-area dependent; fine control of material’s porosity may allow the activity of such materials to be further maximized; and thus, this is of keen interest as a future endeavor. Overall, these materials open new avenues for the successful practical application of enzymes and may have significant medical and industrial utility.

## Supporting Information Available

Materials and methods; (Figure S1) differential scanning calorimetry cycles of solvent-free melts; (Figure S2) synchrotron radiation–wide angle X-ray scattering of solvent-free melts; (Figure S3) synchrotron radiation–circular dichroism spectrum of solvent-free melt; (Figure S4) optical image of annealed solvent-free melt; (Figure S5) G-code schematic; (Figure S6) surface area–observed activity correlation; (Figure S7) scanning electron microscopy micrographs of materials; (Figure S8) optical images of moldable materials; (Figure S9) atomic force microscopy images of materials; (Figure S10) differential scanning calorimetry cycles of materials; (Figure S11) widefield fluorescence microscopy images of enzymatic activity; (Figure S12) longevity of enzymatic activity; (Figure S13); scanning electron microscopy micrographs of cavitation and degradation; and (Figure S14) pH measurements of the storage solution of composite plastics stored in aqueous conditions (PDF)

## Notes

Data are available at the University of Bristol data repository, data.bris, at https://doi.org/10.5523/bris.2db10nbgrq5w621av14h8fs0c6.
