# Supplementary material for: Three-Dimensional Printable Enzymatically Active Plastics
Source: ACS Appl Polym Mater. 2021 Nov 15;3(12):6070–7. doi: 10.1021/acsapm.1c00845 (PMC9376927; doi:10.1021/acsapm.1c00845)
Supplement: Supplementary file 1 — ap1c00845_si_001.pdf [file ap1c00845_si_001.pdf]

3D printable enzymatically active plastics

*William H. Zhang[1], Graham J. Day[1], Ioannis Zampetakis[2], Michele Carrabba[3], Zhongyang Zhang[4], Ben M. Carter[1], Norman Govan[5], Colin Jackson[6,7,8], Menglin Chen[4], Adam W. Perriman[1]**

[1] School of Cellular and Molecular Medicine, University of Bristol, Bristol, BS8 1TD, United Kingdom.

[2] Bristol Composites Institute (ACCIS), University of Bristol, Bristol, BS8 1TR, United Kingdom.

[3] Bristol Medical School, Translational Health Sciences, University of Bristol, Bristol, BS2 8DZ, United Kingdom.

[4] Interdisciplinary Nanoscience Center (iNANO), Aarhus University, Aarhus, DK-8000, Denmark.

[5] Defence Science and Technology Laboratory, Porton Down, Salisbury, SP4 0JQ, United Kingdom.

[6] Australian National University, Research School of Chemistry, Canberra, ACT, 2601, Australia.

[7] Australian Research Council Centre of Excellence for Innovations in Peptide and Protein Science, Research School of Chemistry, Australian National University, Canberra, 2601, ACT, Australia.

[8] Australian Research Council Centre of Excellence in Synthetic Biology, Research School of Chemistry, Australian National University, Canberra, 2601, ACT, Australia.

Corresponding Author

Adam W. Perriman

ORCID: 0000-0003-2205-9364

*E-mail: chawp@bristol.ac.uk.

Supporting Materials and methods

Expression of arPTE and sfGFP

In brief, arPTE (in vector PETMCS3) was transformed into BL21(DE3) *E. coli* cells and expressed without chemical induction in Terrific Broth (TB; Fisher, UK) supplemented with 100 µM cobalt chloride and 50 mg carbenicillin at 25°C for 36–48 hours. The bacteria were then harvested through centrifugation and either purified immediately or the pellets were stored at −20°C for future purification. sfGFP (in the pBAD vector) was into BL21(DE3) *E. coli* cells and in Terrific Broth (TB; Fisher, UK) supplemented with 50 mg carbenicillin at 37°C expressed with arabinose induction at O.D = 0.6. The bacteria were then harvested 4 h after induction through centrifugation and either purified immediately or the pellets were stored at −20°C for future purification.

Purification of arPTE

A bacterial cell pellet (from 500 mL of culture) was resuspended in 25 mL of buffer A (30 mM HEPES, 100 µM cobalt chloride, pH 8.0) and lysed through sonication. The lysate was clarified through high-speed centrifugation and the supernatant was retained. The supernatant was loaded onto a DEAE–Sepharose column (GE Healthcare, UK) that had been pre-equilibrated with buffer A, and the column underwent isocratic elution with buffer A. The fractions containing arPTE were pooled and then concentrated into a small volume (5–10 mL), which was then loaded onto a pre-equilibrated Superdex 200 pg HiLoad 26/600 column (GE Healthcare) and underwent isocratic elution with buffer A. The peak fractions from the size exclusion chromatography (SEC) purification were pooled and was stored at 4°C for future use. The protein after SEC had single band purity as assessed by SDS–PAGE electrophoresis.

Purification of sfGFP

A bacterial cell pellet (from 1 L) was resuspended in 30 mL of buffer B (30 mM HEPES, 200 mM NaCl, 20 mM imidazole, pH 8.0) and lysed through sonication. The lysate was clarified through high-speed

centrifugation and the supernatant was retained. The supernatant was loaded onto a nickel affinity column 10–15× column volumes of buffer B. The protein was then eluted with buffer C (30 mM HEPES, 200 mM NaCl, 250 mM imidazole, pH 8.0) and the fractions containing the protein were retained. The protein was then dialyzed against buffer D (30 mM HEPES, 200 mM NaCl, pH 8.0). The proteins had single band purity as assessed by SDS–PAGE electrophoresis.

Synthesis of oxidized IGEPAL-890

The method for IGEPAL-890 oxidation was followed as previously described by Armstrong *et al.*[1] In brief, IGEPAL-890 (2 g, 1.0 mmol) was dissolved in MilliQ water (50 mL), and Sodium Bromide (50 mg, 0.49 mmol), Sodium Hypochlorite (5 mL, 10–15% available chlorine) and TEMPO (30 mg, 0.19 mmol) was then added to the stirring solution. The solution was basified to pH 11.0 and allowed to stir overnight, after which, the solution was acidified to pH 1.0 and extracted 3 times with chloroform (80 mL each). The chloroform was then washed 3 times with pH 1.0 MilliQ water (80 mL each) and then the chloroform was removed by rotary evaporation. The resulting oil was further purified through recrystallization in neat ethanol at −20°C.

Enzyme conjugation and melt preparation procedure

The amount of surfactant used for conjugation was determined using the following equation:

$$\text{Moles of surfactant} = \text{Moles of enzyme } \times \text{number of charged residues } \times 2.5 \qquad \text{(eq. 1)}$$

With the number of charged residues being the number of anionic residues for the cationic surfactant, and the number of cationic residues for the anionic surfactant. The cationic surfactant (Ethoquad HT 25) was dissolved in the appropriate buffer (A, D, or E. 30 mg/mL) and then rapidly added to a stirring solution of enzyme (100–150 μM). The solution was stirred for 3 hours at which point a solution containing anionic surfactant (oxidized IGEPAL-890), which was prepared in an identical manner to the cationic surfactant, was added and the resulting solution allowed to stir for 3 hours or overnight. The solution then underwent dialysis with MilliQ water for 16–24 hours with one

change of the external dialysis solution at 3 hours. The solution was then lyophilized, and the resulting powder was thermally annealed (45°C) to create the enzyme melt. In this liquid form, the melt can be dissolved in warm organic solvents (45°C). The presence of water, either due to ambient exposure or inadequate lyophilization, can prevent the melt from correctly dissolving in organic solvents and can result in turbidity.

Synchrotron Radiation Wide Angle X-ray Scattering (SR–WAXS) analysis of melt

The data for the [arPTE][S^+][S^-] melt was obtained using synchrotron radiation–wide angle X-ray scattering (SR–WAXS) on the I22 beamline at the Diamond Light Source, Oxford. Samples were loaded into punctured DSC pans lined with capton film, and 1 frame was recorded for 1 second per 1°C, heating at 1°C a minute. Data preparation and reduction to generate the 2D scattering profiles were performed using DAWN.

Synchrotron Radiation–Circular Dichroism (SR–CD) analysis of melt

The data for the [arPTE][S^+][S^-] melt was obtained using synchrotron radiation-circular dichroism (SR–CD) B23 beamline at the Diamond Light Source. A thin film of the melt was held between two quartz slides, and the CD spectrum was collected from 180–260 nm with one spectrum collected per 3°C, heating at 1°C a minute.

Piston-Driven 3D (PD3D) printing

Piston-driven 3D printing consists in the extrusion of a wide range of acellular viscous inks or hydrogel from the nozzle of a syringe guided by a piston with an accurate resolution. The MendelMax 3.0 3D printer (Maker's Tool Works, US) was retrofitted with a custom extruder able to drive a 5 mL syringe. The printing of the solution is driven by the motorized piston, composed by a NEMA17 stepper motor connected with a metallic piston that moves the plunger of the syringe, ensuring a controlled deposition of the polymeric solution.

This approach allows the fabrication of the complex structure by a continuous deposition of the material from the nozzle of the syringe to the stage. The 3-dimensionality of the structures is achieved by a layer-by-layer extrusion of the polymeric solution, in which the deposition of the first layer is followed by a period of stabilization to allow the evaporation of the volatile solvent, before the extrusion of the second layer.

A low viscous solution of PCL was dissolved in high volatile solvent and printed through jetting and extruding with high resolution as previously described.[2] In brief, PCL (average MW 80,000; Sigma Aldrich) solution was prepared dissolving the polymer in chloroform at 20% (w/v) and the conjugated enzymes were added at 1% (w/w). The solution of PCL–conjugated enzyme was then loaded into the 5 ml syringe and mounted on the extruder driven by the motorized piston. The simple geometry of a ring was chosen to characterize the enzymatic activity in aqueous environment at different timepoints. To test the correlation between the activity and physical features, 4 rings were fabricated (**Figure S4**): i) 1 layer, 21 rings; ii) 3 layers, 16 rings; iii) 5 layers, 11 rings; iv) 7 layers, 7 rings. The motion speed of the piston was set-up at 0.2 mm·min^{-1} and a 27-gauge nozzle was used.

The feasibility of fabricating a PCL–conjugated enzyme structure with higher complexity was successfully demonstrated by printing a woodpile. The structure consists in the overlap of 6 layers of grid in order form a 3D matrix. For this experiment, a 30-gauge nozzle was used and the speed of the piston 0.15 mm·min^{-1} was set-up achieving a line width of 100 µm. The use of our composite material with this printing technique follows the same technical and structural capabilities and restrictions as described previously.[2]

Filament extrusion & thermal moulding procedure

PCL (average MW 80,000; Sigma Aldrich) was initially dissolved in chloroform and the conjugated enzymes were added in the mixture at 1% w/w conjugate to PCL. The mixture was then left to dry for the chloroform to completely evaporate overnight. The resulting enzyme–PCL hybrid material was

processed into fine pieces and fed through a Noztek Pro extruder at 70°C and a filament of diameter varying between 1.5–1.7 mm was generated. The filament was then used for thermal moulding of ring specimens. An aluminium mould was used, around which the filament was wrapped to generate the ring shape, which was kept at 60°C for 1 hour for the ring specimen to mould. The specimens were left to cool for one hour and they were then removed from the mould for further testing.

Melt Electrowriting (MEW) procedure

PCL (average MW 45,000; Sigma Aldrich) was initially dissolved in chloroform and conjugated enzyme or sfGFP were added in the mixture at 0.1% w/w and 0.2% w/w conjugate to PCL. The mixture was then left to dry for the chloroform to completely evaporate overnight. The enzyme plastics were loaded into a stainless-steel syringe of a melt electrowriting instrument (CAT000111, Spraybase), which was equipped with a 24 G blunt-end spinneret. A gas pressure of 0.15 bar was supplied to extrude the polymer melt out from the spinneret at 70°C, while the needle was located at 4 mm away from the collector. A high voltage of 4 kV was applied between the collecting plate and the spinneret, generating a strong electrostatic field that leads to the formation of Taylor cones in the spinneret tip and stretches the melt into thinner jet. By controlling movement (including velocity, orientation) of the collector *via* UCCNC software, various patterned fabrics composed of either straight or coiled fibres could be manufactured. Running velocity of 200 mm/min and 5000 mm/min was used in our study for tangles and straight lines, respectively. The percentage loading of enzyme in the material can affect the viscosity of the material, and thus the optimization of printing parameters is necessary when using printing with composites of differing enzyme loading percentages.

Paraoxon assay of arPTE activity

Assays of the [arPTE][S^+][S^-]–PCL material monitored the hydrolysis of the simulant paraoxon through absorbance at 405 nm.[3] Plastic of a known weight and enzyme loading was placed in a buffered solution (30 mM HEPES, 200 mM NaCl, pH 8.0) and the reaction was initiated through the

addition of the paraoxon. The solution was shaken briefly for mixing immediately prior to an absorbance reading, and the derived activity from the reaction used the assumption that the total enzyme present was homogenously distributed throughout the solution, without correcting for the surface-restricted reaction interface of the material.

For the time-course studies of the material for reusability and activity persistence, the material was left in a solution of buffer (30 mM HEPES, 200 mM NaCl, pH 8.0) in between measurements to allow for environmental degradation, if any, of the PCL by water, and to ensure that the material was constantly exposed to an environment where the enzyme could potentially be depleted.

Scanning Electron Microscopy (SEM) analysis of plastics

Samples analysed on a FEI Quanta 200 field emission gun-SEM (ThermoFisher Scientific, USA) under high-vacuum. A beam energy of 10.0 kV was used and the SEM equipment was accessed through the Wolfson Bioimaging Facility, Faculty of Life Sciences, University of Bristol. Samples were analysed without a coating unless specified. Coated samples were sputter coated with a 15 nm gold/palladium layer.

Differential Scanning Calorimetry (DSC) analysis of plastics

Samples were analysed on a DSC Q100 (TA instruments), using 5–10 mg of sample (either solid plastic, or the cooled solidified protein melt) in aluminium non-hermetic pans unless otherwise specified, with an empty pan as the reference sample. The samples, starting at 40°C, were heated to 80°C and then cooled to −80°C. This was then cycled three times between 80°C and −80°C at a heating/cooling rate of 10°C/min, and the resulting observed changes in heat flow allowed for the determination of crystallization transition temperature and the melting transition temperature.

Atomic Force Microscopy (AFM) analysis

The electrospun fibres of PCL, [sfGFP][S$^+$][S$^-$]–PCL, and [arPTE][S$^+$][S$^-$]–PCL (0.1% w/w, PCL MW 45,000) were prepared on a silicon dioxide substrate and characterized by using a Dimension ICON

AFM (Bruker, Santa Barbara, CA, USA) with a chamber temperature of 22.5°C and humidity of 33%. A TAP150A probe (resonance frequency: 150 kHz, spring constant: 5 N·m^{-1} and tip radius: 8 nm; Bruker, USA) was used to conduct Peakforce QNM (Quantitative Nanomechanics) mapping to characterize the morphology and modulus of fibre surfaces. The images were obtained under a scan rate of 0.977 Hz and a scan resolution of 512 × 512 pixels. Morphology images, and statistical analysis was performed by using SPIP software (Image Metrology ApS, Lyngby, Denmark).

Aqueous sample pH measurements

1 cm^2 samples of 0.1% w/w [sfGFP][S^+][S^-]–PCL and [arPTE][S^+][S^-]–PCL were incubated in 5 mL pure dH_2O and pH measurements were recorded every 24 h for 7 days.

Supporting tables

Enzyme state	Activity (k_{cat}; s^{-1})
Unmodified arPTE	267 ± 12 s^{-1} [ref. [4]]
[arPTE][S+][S-]	575 ± 23 s^{-1}[ref. [4]]
[arPTE][S^+][S^-]–PCL (1%)	0.54 ± 0.02

Table S1: A table comparing the observed k_{cat} between the unmodified enzyme, surfactant conjugated enzyme, and the enzyme-plastic composite. As [arPTE][S^+][S^-]–PCL is not freely diffusing as expected under the Michaelis-Menten model of enzyme kinetics, the observed k_{cat} of [arPTE][S^+][S^-]-PCL should not be directly compared with the free enzyme.

Supporting figures

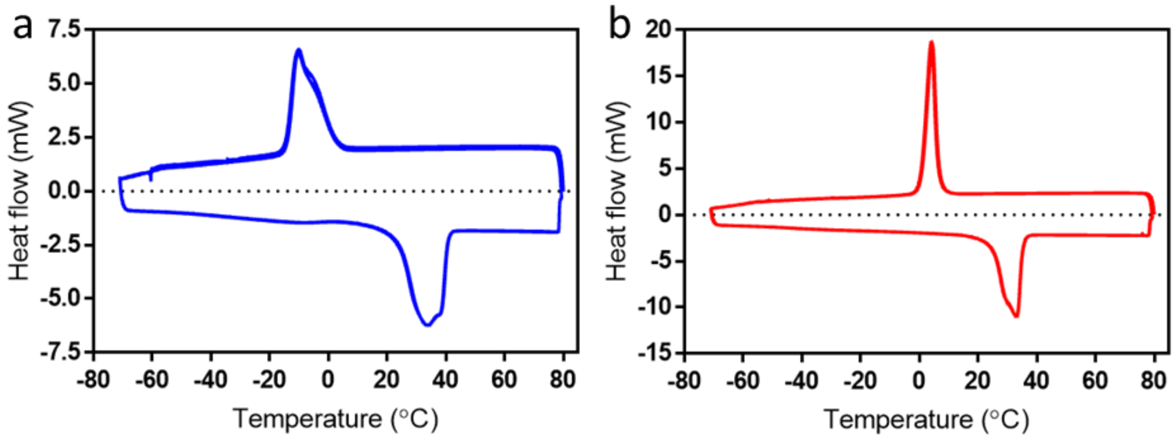

Figure S1: a) differential scanning calorimetry cycles of the [arPTE][S^+][S^-] melt with the initial DSC dummy run removed with the crystallization transition at −10.3 ± 0.2°C and the melting transition at 33.0 ± 0.1°C. b) Cycle of the [sfGFP][S^+][S^-] melt, likewise with the initial dummy run removed, with the crystallization transition at 4.2 ± 0.2°C and the melting transition at 32.96 ± 0.04°C. Samples were analysed using aluminium non-hermetic pans. The temperature cycling protocol was as described in the methods.

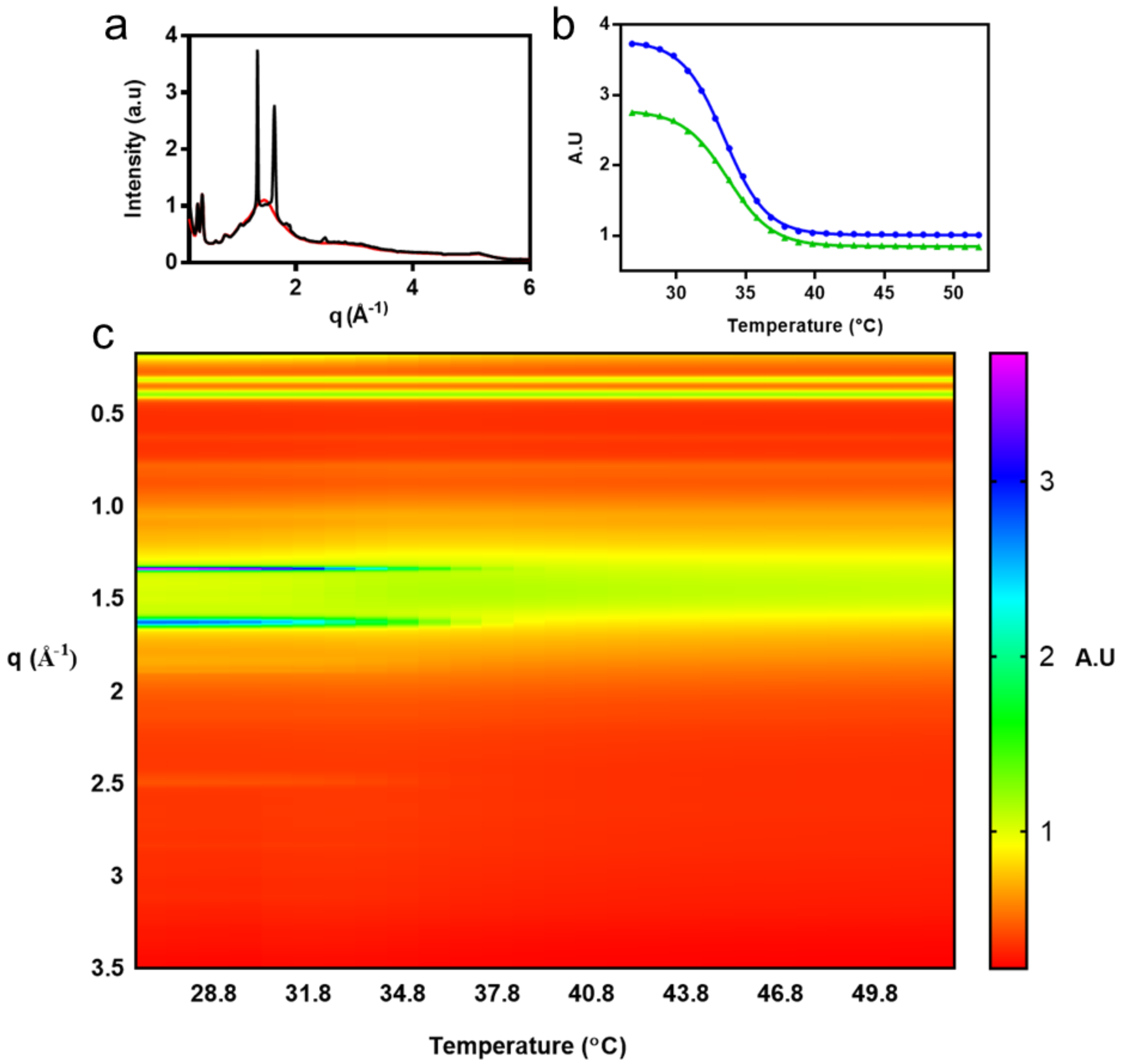

Figure S2: a) The synchrotron radiation–wide angle x-ray scattering spectra at room temperature (black) compared to a melted sample (red, 45°C) showing a loss of crystalinity as the melt becomes a liquid. b) Graph displaying the loss of intensity as a function of temperature at *q* = 1.339 (blue) and 1.630 (green), with the melt becoming an amorphous liquid completely at 41°C. c) The spectra as a 2D graph as a function of temperature.

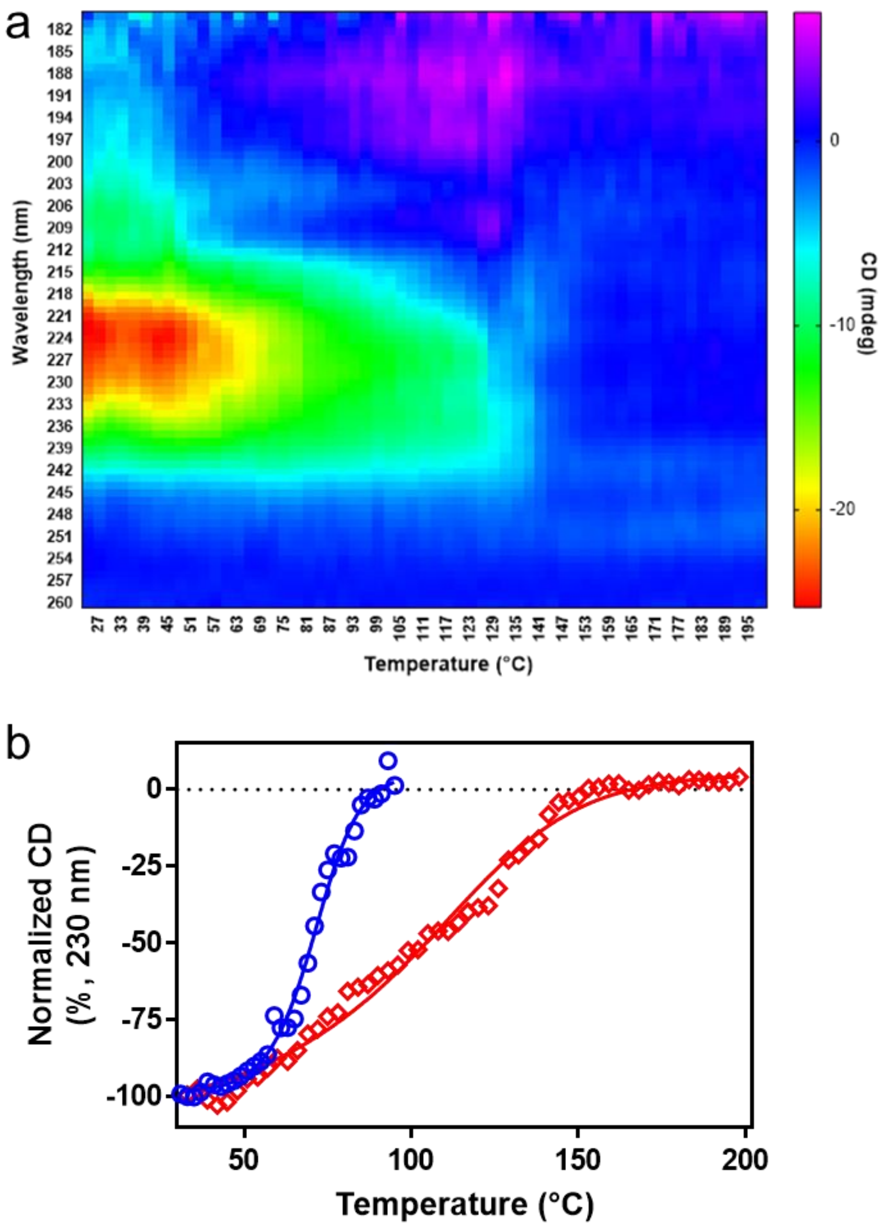

Figure S3: a) The synchrotron radiation–circular dichroism spectrum of the [arPTE][S$^+$][S$^-$] melt as a function of temperature, with the secondary structure of the material (indicative of folded enzyme) only fully disappearing at approximately 147°C. b) The absorbance at 230 nm as a function of temperature of the unmodified arPTE in aqueous solution (blue) compared to the [arPTE][S$^+$][S$^-$] melt showing that the melt has an enhanced thermostability.

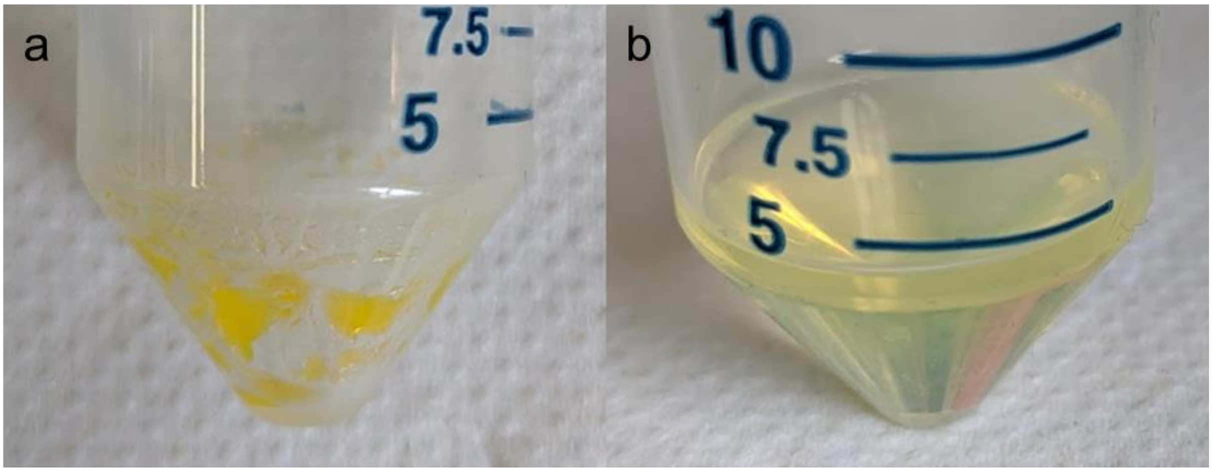

Figure S4: a) The [sfGFP][S$^+$][S$^-$] melt as a solid after the lyophilized powder was thermal annealed to the solvent-free melt, then allowed to cool. b) The [sfGFP][S$^+$][S$^-$] melt after heating the melt back into a viscous liquid state followed by dissolution into dry warm (45 °C) chloroform. The solubility of the melted material is at least 50 mg/ml, and dissolves best with warm solvent. Room temperature solvent may result in less dissolution of the material.

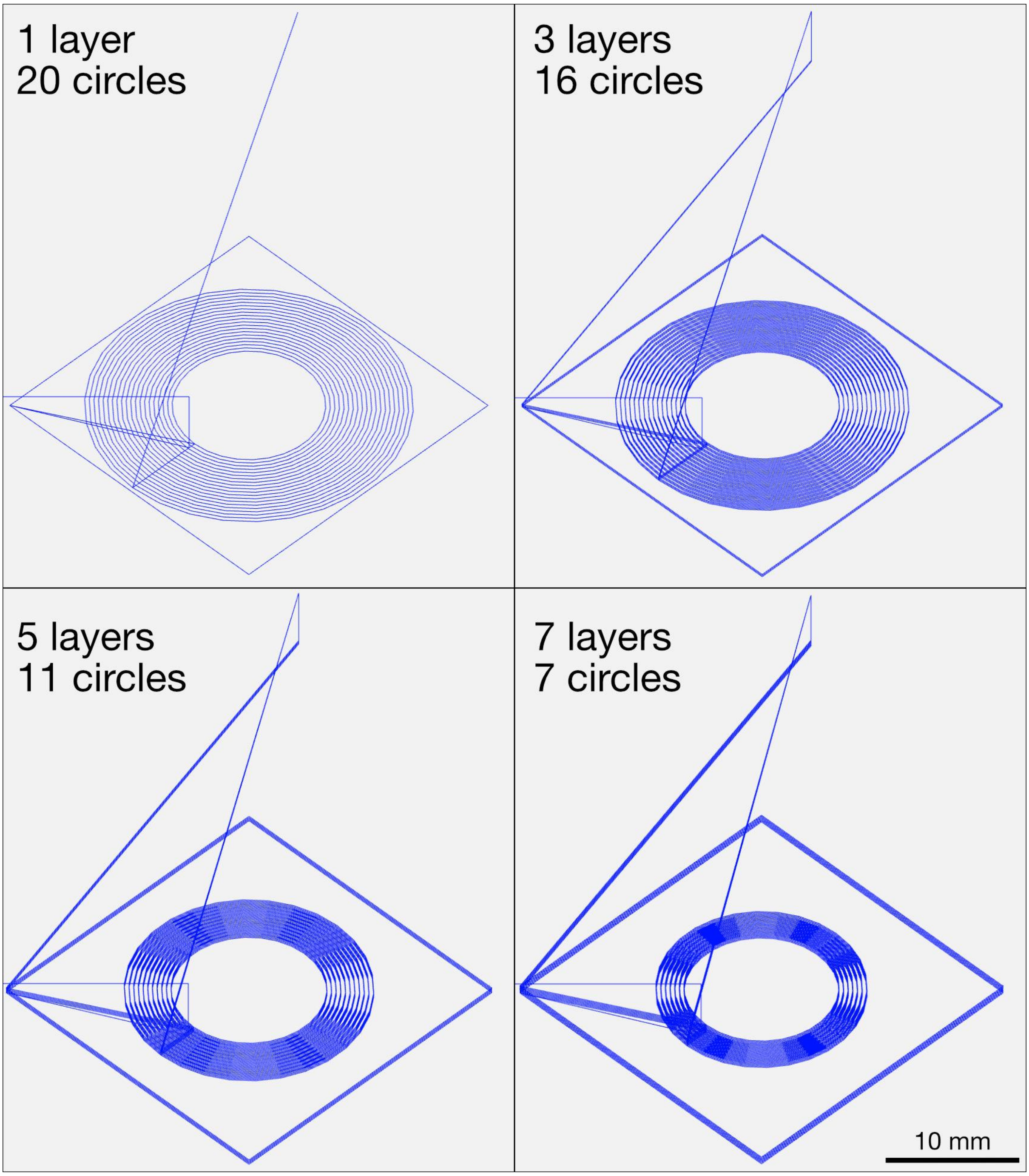

Figure S5: Schematic derived from the G-code depicting the printing pathway to generate the multi-layer rings. Diameters were varied in order to control for the total ring mass and in order to better ensure that the tested variables were surface area and layer number.

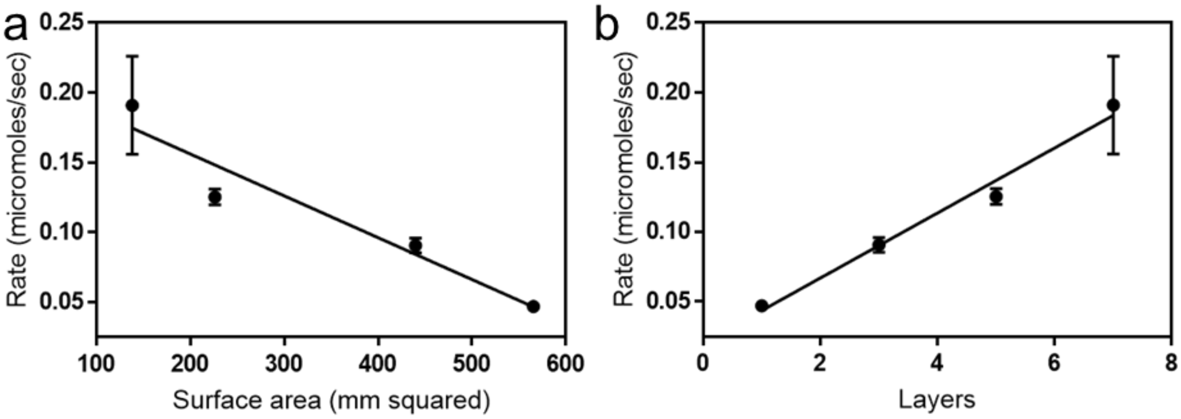

Figure S6: a) A negative correlation between the observed activity and the surface area of the printed disk (without accounting for internal surface area), which would superficially suggest that the smaller the surface area, the more catalytic the fabricated object. b) The positive correlation with layer number and the observed activity is consistent with the inter-layer surface area being substantially greater than that of the exposed surfaces.

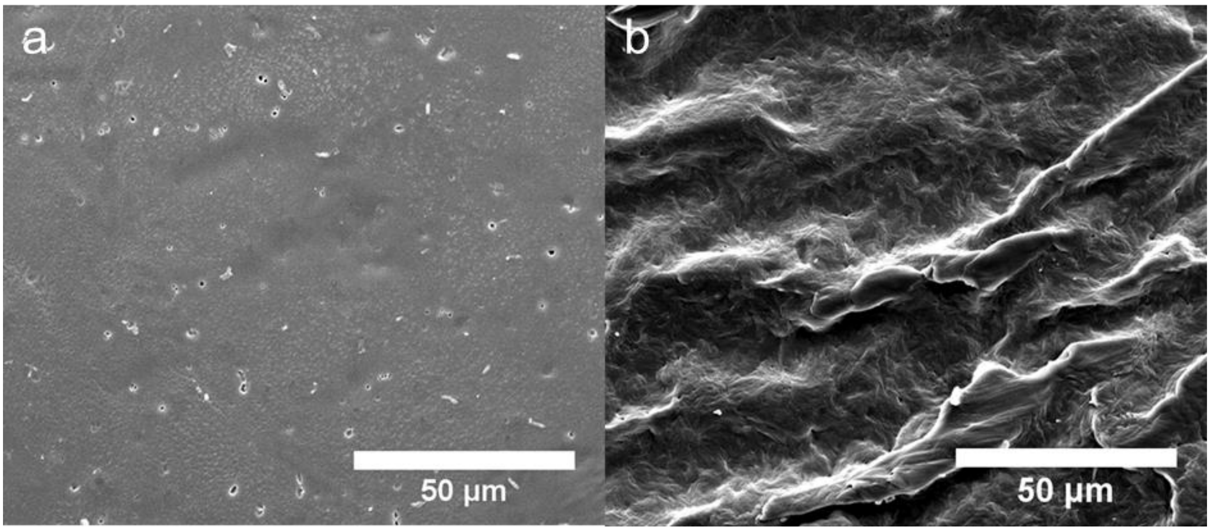

Figure S7: a) Scanning electron microscopy of the exposed external surface showing relative smoothness (and therefore low surface area) of the printed multi-layer PCL rings. Cavities are visible as a sign of degradation due to extended exposure to an aqueous environment. b) the inter-layer surface area showing a wrinkled surface and irregular features, which would greatly increase the effective surface area of the layer. Both samples were coated prior to analysis and were analysed as described in the supporting methods.

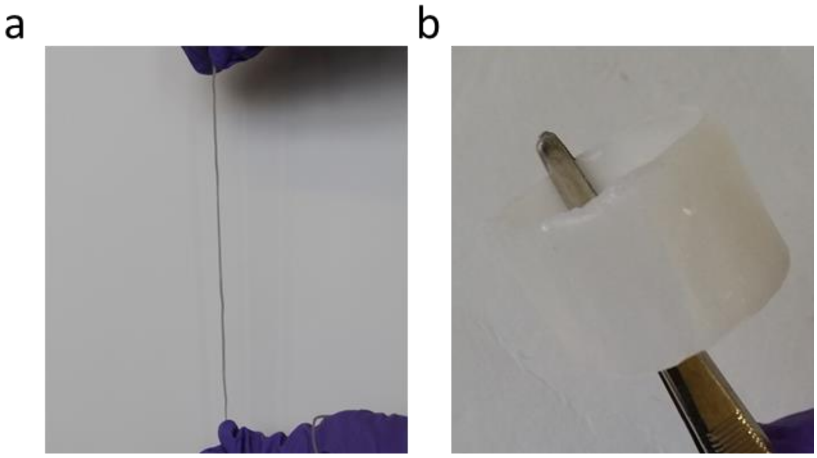

Figure S8: a) The [arPTE]$[S^+][S^-]$–PCL material after conversion into a 3D-printable filament, with trace amounts of black colourant present. The cord is, in principle, compatible with many standard cord-based 3D printers. b) the bulk material can be melted and moulded directly by hand at 60°C without macroscale precision to create larger structures.

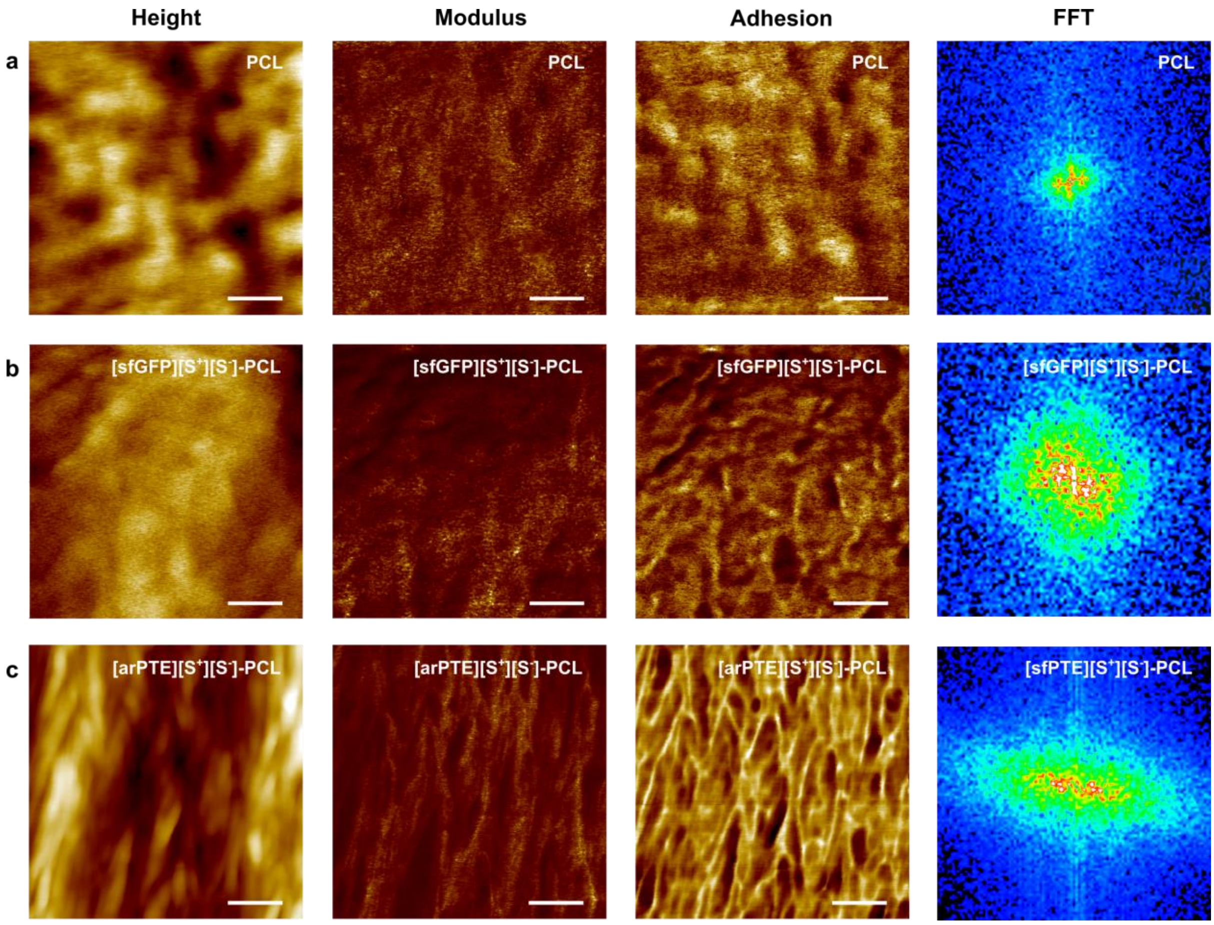

Figure S9: Topographical morphology, Young's modulus, adhesion and FFT analysis of melt electrowritten fibres from AFM analysis of a) PCL, b) [sfGFP][S^+][S^-]–PCL, and c) [arPTE][S^+][S^-]–PCL. Scale bar 200 nm. Compared to PCL, [arPTE][S^+][S^-]–PCL shows obvious fibre alignment, and significantly increased Young's modulus.

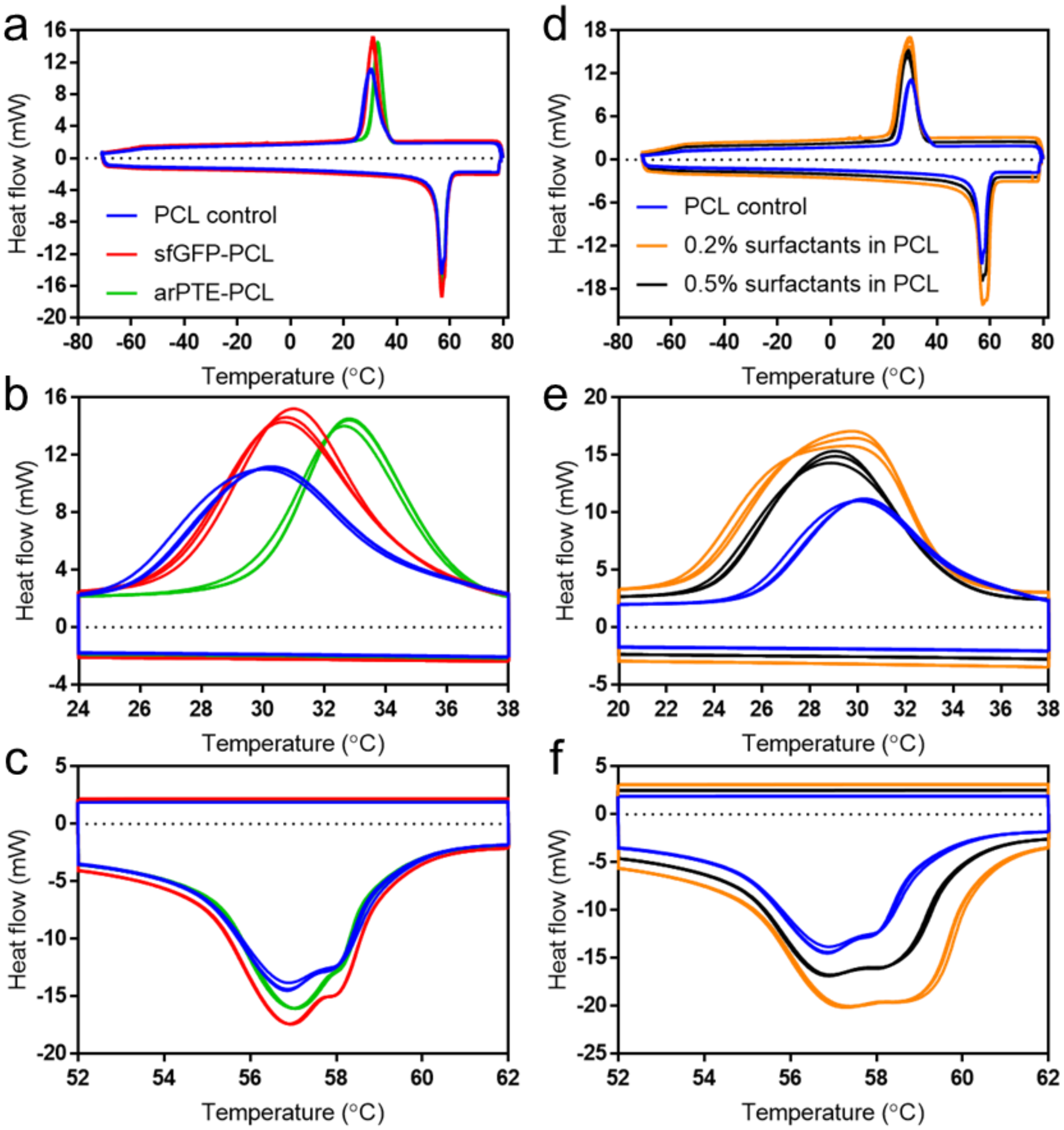

Figure S10: a) Differential scanning calorimetry cycles of PCL (blue), [sfGFP]$[S^+][S^-]$–PCL (0.1% protein w/w, red), and [arPTE]$[S^+][S^-]$–PCL (0.1% protein w/w, green) with b) the crystallization transition of the materials (30.2 ± 0.1°C for PCL, 30.8 ± 0.2°C for [sfGFP]$[S^+][S^-]$–PCL, and 32.7 ± 0.1°C for [arPTE]$[S^+][S^-]$–PCL) and c) the melting transitions (56.87 ± 0.02°C for PCL, 56.92 ± 0.03°C for [sfGFP]$[S^+][S^-]$–PCL, and 57.03 ± 0.03°C for [arPTE]$[S^+][S^-]$–PCL), with the initial DSC dummy run removed. The overlay of the samples show that the crystallization transition temperature shifts depending on the identity of the protein incorporated into the plastic, while there does not appear to be the same corresponding shift to the melting temperature of the material. d) Cycles of PCL containing the appropriate surfactants showing that the presence of the surfactants is not responsible for the increase in the crystallization transition temperature as shown in e) where we see that the surfactant doped material has a slight decrease in the crystallization temperature with increasing surfactant (29.8 ± 0.1°C for 0.2% surfactant–PCL and 29.0 ± 0.1°C for 0.5% surfactant). f)

Conversely, we see that the melting transition is shifted asymmetrically towards a higher temperature, although the peak melting transition does not change significantly (57.3 ± 0.1°C for 0.2% surfactant–PCL and 56.91 ± 0.01°C for 0.5% surfactant–PCL). As with the solvent-free enzyme melts, all samples were analysed using aluminium non-hermetic pans. The temperature cycling protocol for DSC analysis was as described in the methods.

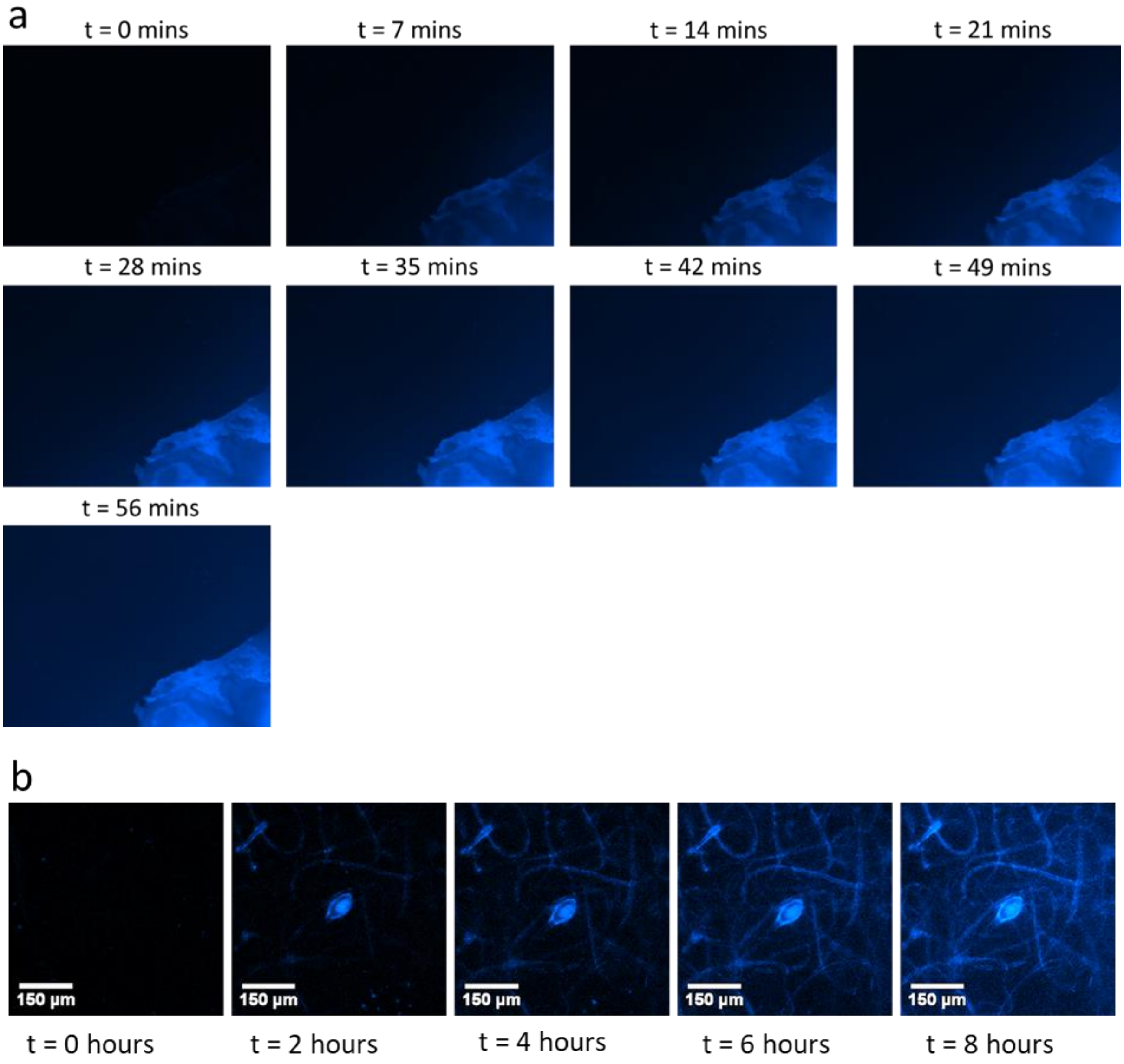

Figure S11: a) A time lapse of the bulk [arPTE]$[S^{+}][S^{-}]$–PCL material (0.5% enzyme w/w) in a solution of Coumaphos (1 mM). The material becomes increasingly fluorescent, however the surrounding solution only increases in fluorescence after the fluorescence of the material has begun to plateau. This is consistent with the reaction occurring at the interface between solution and material, and the product and substrate exchange slowly through diffusion. b) A fibre network of [arPTE]$[S^{+}][S^{-}]$–PCL (0.1% enzyme w/w) printed using thermal extrusion (melt electrowriting) similarly shows hydrolysis of Coumaphos, where the fibres and surface of the material exhibit activity, accumulating the fluorescent product. Excitation wavelength = 358 nm.

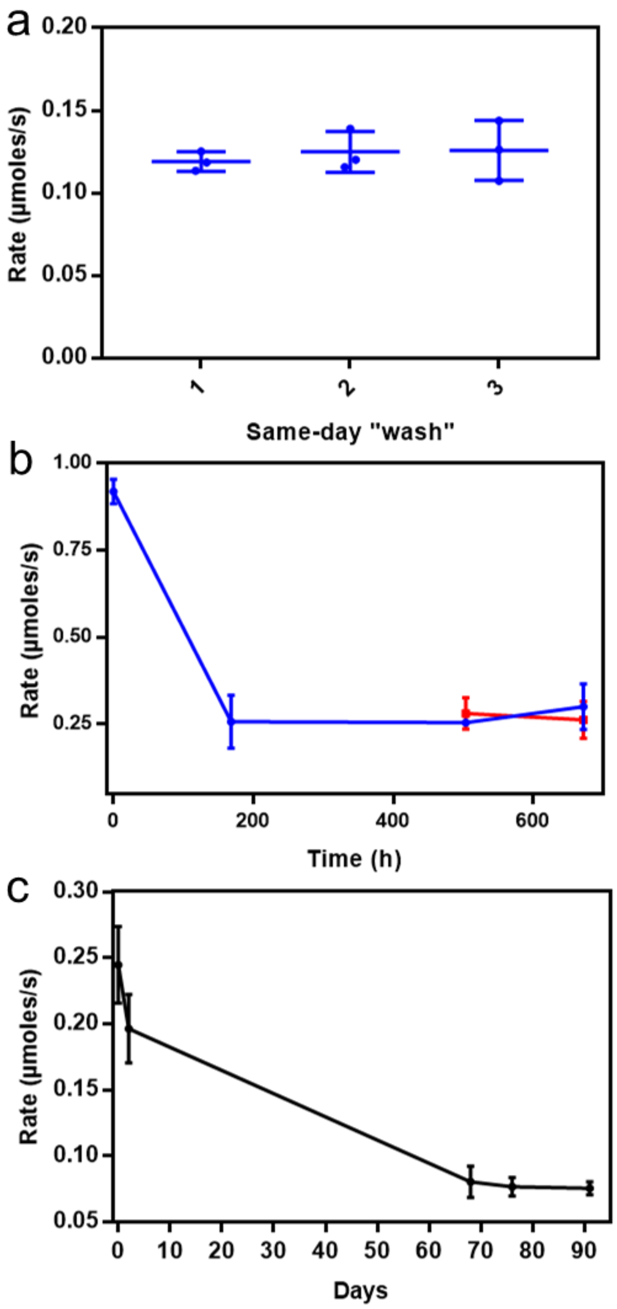

Figure S12: a) The observed rate for [arPTE][S^+][S^-]–PCL rings printed using PD3D printing assayed in succession, with rinse steps between assays. There is no statistically significant difference between the rates of the assays suggesting that a decline in activity is not due to enzyme liberation. b) Assays of [arPTE][S^+][S^-]–PCL rings that have been assayed in solution, and stored in solution between assays, across a long time period compared to identical disks held in dry storage. As there the decline in activity under both conditions reaches the same point, the cause is most likely to be time related aging of (some population) of the enzyme. c) Rings stored even longer retain activity at 3 months (91 days) after fabrication, all while being persistently in aqueous solution.

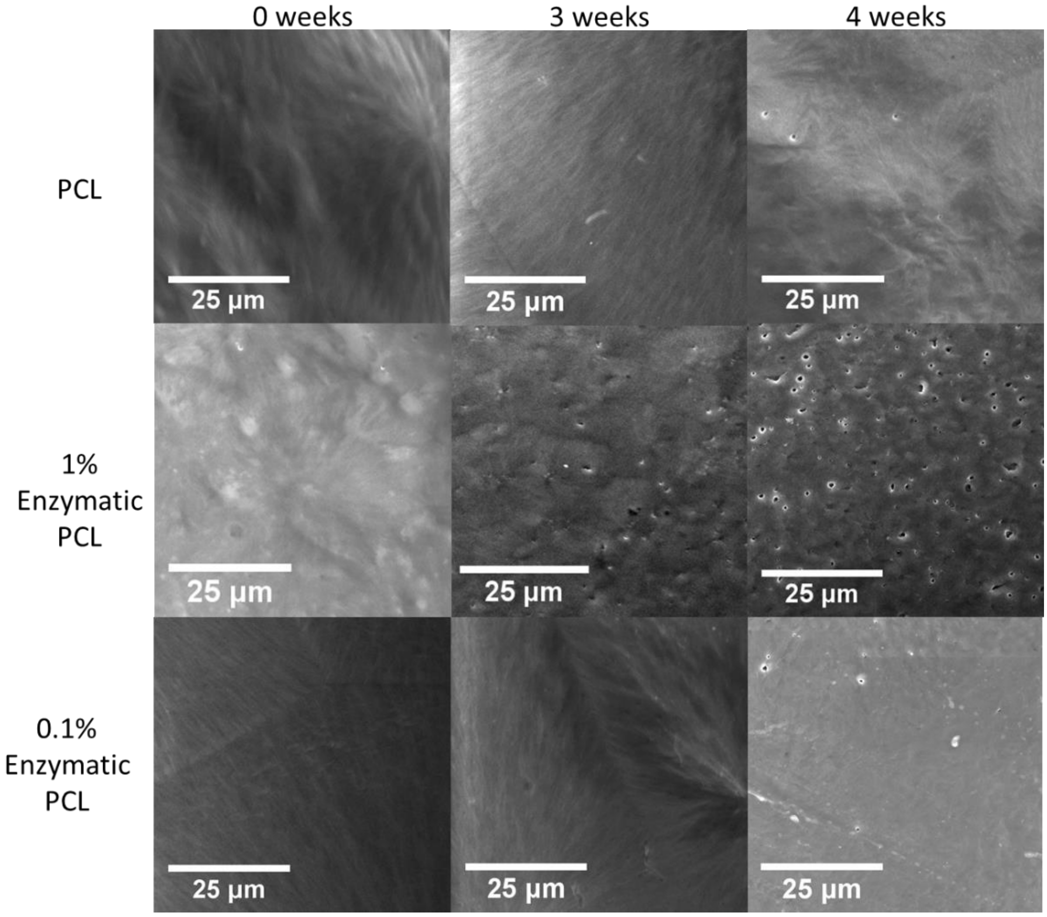

Figure S13: Scanning electron microscopy images showing the relationship between enzyme loading into the PCL and the rate of cavitation/degradation. 1% enzyme–PCL (w/w) shows a greater degree degradation when compared to 0.1% enzyme–PCL or enzyme-free PCL. Conversely, 0.1% enzyme–PCL has a comparable level of degradation to the control enzyme, showing that at this level of enzyme loading it should not affect the structural integrity of the material, at least in terms of environmental degradation. It should be noted that the cavities shown in this figure in the 0.1% enzyme–PCL and the control are only to serve as an example of cavitation, and that these cavities are otherwise rare and are only sparsely observed for the overall sample. Samples were not coated for SEM analysis at any stage and were analysed as described in the supporting information.

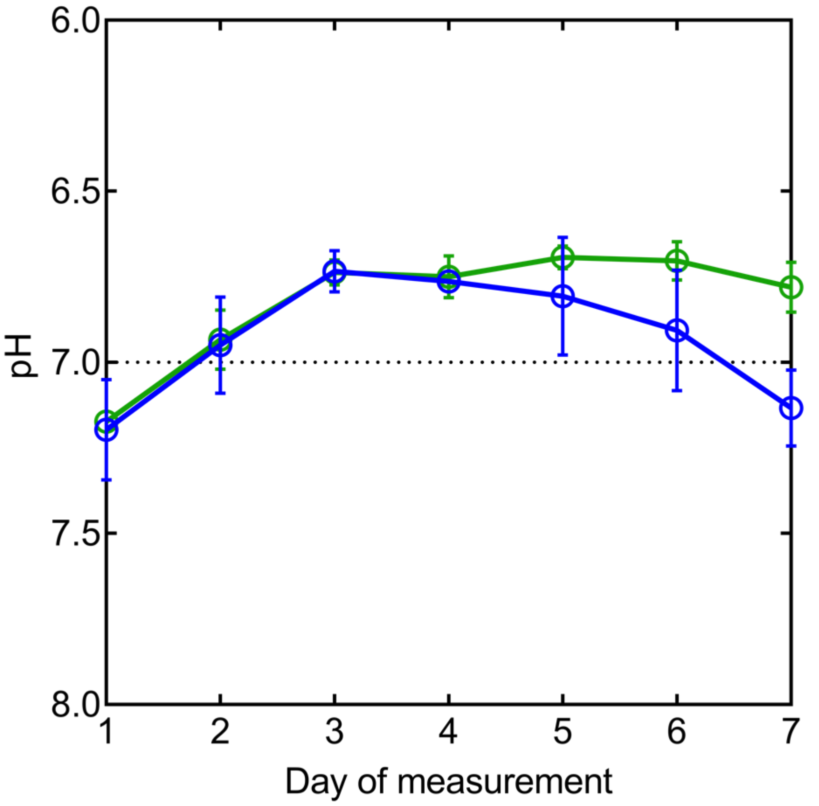

Figure S 14: pH measurements of 0.1% enzyme in PCL stored under aqueous conditions. The average pH measurements of [sfGFP][S^{+}][S^{-}]–PCL (green), and [arPTE][S^{+}][S^{-}]–PCL (blue), stored in pure water for seven days. Error bars are standard deviation, n = 3.

References

1. Armstrong, J. P. K., Burke, M., Carter, B. M., Davis, S. A. & Perriman, A. W. 3D Bioprinting Using a Templated Porous Bioink. *Adv. Healthc. Mater.* **5**, 1724–1730 (2016).

2. Carrabba, M. *et al.* Design, fabrication and perivascular implantation of bioactive scaffolds engineered with human adventitial progenitor cells for stimulation of arteriogenesis in peripheral ischemia. *Biofabrication* **8**, 015020 (2016).

3. Donarski, W. J., Dumas, D. P., Heitmeyer, D. P., Lewis, V. E. & Raushel, F. M. Structure-activity relationships in the hydrolysis of substrates by the phosphotriesterase from Pseudomonas diminuta. *Biochemistry* **28**, 4650–5 (1989).

4. Zhang, W. H. *et al.* Sequential Electrostatic Assembly of a Polymer Surfactant Corona Increases Activity of the Phosphotriesterase arPTE. *Bioconjug. Chem.* **30**, 2771–2776 (2019).
